# Pericardial bubbles: unraveling an iatrogenic pericardial effusion

**DOI:** 10.1093/ehjcr/ytaf194

**Published:** 2025-04-17

**Authors:** Francesco Moda, Elisa Gherbesi, Guido Gelpi, Stefano Carugo

**Affiliations:** Department of Clinical Sciences and Community Health, University of Milan, Via Festa del Perdono 7, 20122 Milan, Italy; Department of Cardio-Thoracic-Vascular Diseases, Foundation IRCCS Ca’Granda Ospedale Maggiore Policlinico, Via Francesco Sforza 35, 20122 Milan, Italy; Department of Cardio-Thoracic-Vascular Diseases, Foundation IRCCS Ca’Granda Ospedale Maggiore Policlinico, Via Francesco Sforza 35, 20122 Milan, Italy; Department of Clinical Sciences and Community Health, University of Milan, Via Festa del Perdono 7, 20122 Milan, Italy; Department of Cardio-Thoracic-Vascular Diseases, Foundation IRCCS Ca’Granda Ospedale Maggiore Policlinico, Via Francesco Sforza 35, 20122 Milan, Italy

## Case presentation

A 64-year-old woman, 10 days post-kidney transplant and undergoing dialysis via a step-tip central venous catheter, presented with chest discomfort and retrosternal ‘freezing’ sensations triggered by catheter flushes.

On examination, she exhibited muffled heart sounds, weak arterial pulse, and blood pressure of 102/60 mmHg. Electrocardiogram showed sinus tachycardia (104 b.p.m.) with mild PR segment depression, suggestive of pericardial irritation. Chest X-ray revealed no pathological findings and showed the catheter tip projected within the right atrium. Point-of-care ultrasound revealed a significant pericardial effusion.

She was receiving immunosuppressive therapy and prophylactic ceftriaxone post-transplant. Three dialysis sessions had been performed for delayed graft function without complications. The absence of flu-like symptoms, negative viral PCR, normal urea levels, and preserved mental status ruled out infectious or uremic pericarditis. Given the new-onset effusion, she was admitted to the cardiac intensive care unit.

**Figure 1:**
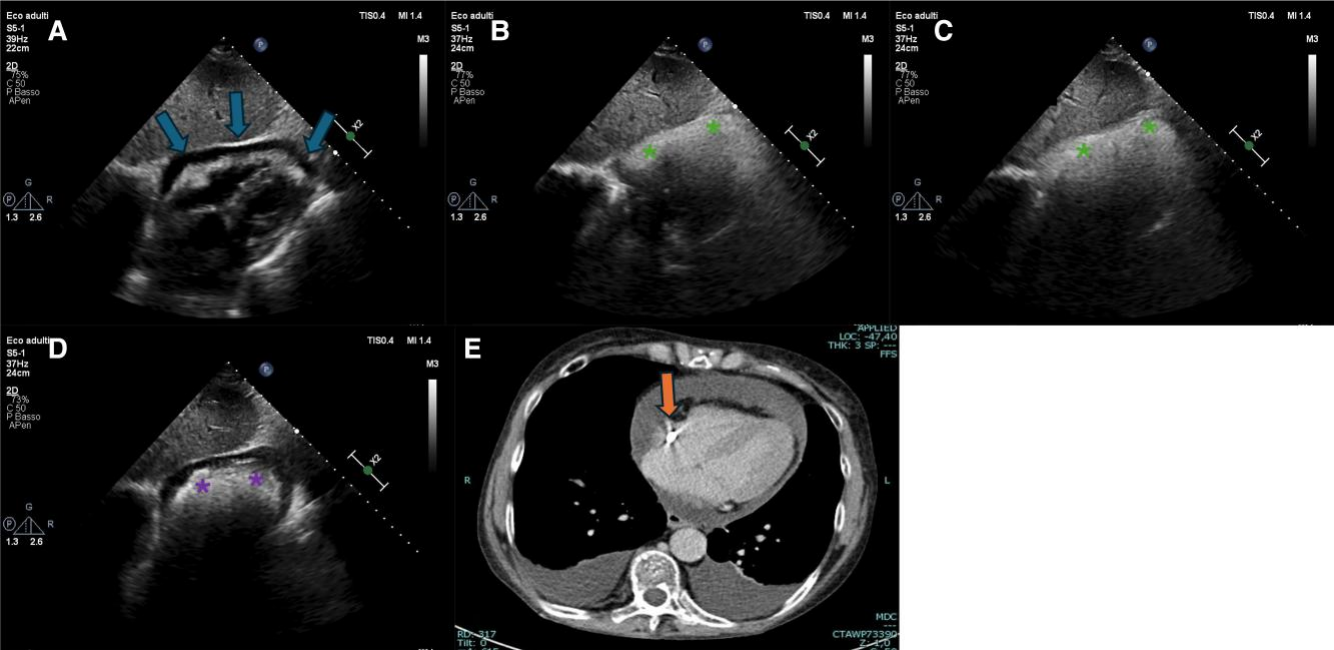
(*A*) Subcostal view showing circumferential pericardial effusion (arrow annotations). (*B*, *C*) Subcostal view showing bubbles in pericardial cavity (star symbols) after contrast injection from the middle and the distal lumens respectively (see Supplementary material online, *Videos S1* and *S2*). (*D*) Subcostal view showing bubbles in right chambers (star symbols) after contrast injection from the proximal lumen (see Supplementary material online, *Video S3*). (*E*) Computed tomography scan showing the catheter tip’s location against the right atrial wall (arrow annotation).

A comprehensive echocardiographic assessment confirmed a circumferential pericardial effusion (1.8 cm in diastole), without haemodynamic compromise^1^ (*Figure 1A*). To investigate the aetiology, a catheter flush test was performed, reproducing symptoms in two of the three lumens, prompting a bubble contrast echocardiogram. Contrast injection through the middle and distal lumens resulted in microbubbles within the pericardial cavity (*Figure 1B* and *C*; Supplementary material online, *Videos S1* and *S2*), whereas contrast injected via the proximal lumen entered the right heart chambers (*Figure 1D*; Supplementary material online, *Video S3*).

These findings confirmed an iatrogenic pericardial effusion secondary to catheter malposition. Chest computed tomography (CT) scan subsequently confirmed right atrial wall perforation (*Figure 1E*), likely tamponaded by the catheter itself. Given procedural risks, catheter removal was performed in a hybrid operating room under cardiothoracic supervision.

The patient recovered uneventfully and was discharged 6 days later.

This case highlights the critical role of contrast-enhanced echocardiography in detecting catheter-related complications,^2^ allowing early diagnosis and intervention. Integrating contrast echocardiography into standard evaluation protocols for central venous catheters can improve early recognition of mechanical complications, enhancing patient safety.

Moreover, a multimodal imaging strategy, including CT, provides complementary anatomical confirmation, guiding precise clinical decision-making and ensuring optimal patient management.

## Supplementary Material

ytaf194_Supplementary_Data

## Data Availability

The data underlying this article are available in the article and in its online Supplementary material.

## Notes

**Consent:** The authors confirm that explicit consent has been obtained from the patient for the publication of medical information included in this article.

**Funding:** This research did not receive any specific grant from funding agencies in public, commercial, or not-for-profit sectors.
